# Delivery of mRNA Vaccine against SARS-CoV-2 Using a Polyglucin:Spermidine Conjugate

**DOI:** 10.3390/vaccines9020076

**Published:** 2021-01-21

**Authors:** Larisa I. Karpenko, Andrey P. Rudometov, Sergei V. Sharabrin, Dmitry N. Shcherbakov, Mariya B. Borgoyakova, Sergei I. Bazhan, Ekaterina A. Volosnikova, Nadezhda B. Rudometova, Lyubov A. Orlova, Inna A. Pyshnaya, Boris N. Zaitsev, Natalya V. Volkova, Mamedyar Sh. Azaev, Anna V. Zaykovskaya, Oleg V. Pyankov, Alexander A. Ilyichev

**Affiliations:** 1 State Research Center of Virology and Biotechnology “Vector”, Koltsovo, 630559 Novosibirsk, Russia; rudometov_ap@vector.nsc.ru (A.P.R.); sharabrin_sv@vector.nsc.ru (S.V.S.); scherbakov_dn@vector.nsc.ru (D.N.S.); borgoyakova_mb@vector.nsc.ru (M.B.B.); bazhan@vector.nsc.ru (S.I.B.); volosnikova_ea@vector.nsc.ru (E.A.V.); andreeva_nb@vector.nsc.ru (N.B.R.); orlova_la@vector.nsc.ru (L.A.O.); zaitsev@vector.nsc.ru (B.N.Z.); tasha_wolkowa11.93@mail.ru (N.V.V.); mamedyar50@mail.ru (M.S.A.); zaykovskaya_av@vector.nsc.ru (A.V.Z.); pyankov_ov@vector.nsc.ru (O.V.P.); ilyichev@vector.nsc.ru (A.A.I.); 2 Institute of Chemical Biology and Fundamental Medicine, Siberian Branch, Russian Academy of Sciences, 630090 Novosibirsk, Russia; pyshnaya@niboch.nsc.ru

**Keywords:** SARS-CoV-2, RBD, mRNA-vaccine, polyglucin:spermidine conjugate, anti-RBD antibodies

## Abstract

One of the key stages in the development of mRNA vaccines is their delivery. Along with liposome, other materials are being developed for mRNA delivery that can ensure both the safety and effectiveness of the vaccine, and also facilitate its storage and transportation. In this study, we investigated the polyglucin:spermidine conjugate as a carrier of an mRNA-RBD vaccine encoding the receptor binding domain (RBD) of the SARS-CoV-2 spike protein. The conditions for the self-assembling of mRNA-PGS complexes were optimized, including the selection of the mRNA:PGS charge ratios. Using dynamic and electrophoretic light scattering it was shown that the most monodisperse suspension of nanoparticles was formed at the mRNA:PGS charge ratio equal to 1:5. The average hydrodynamic particles diameter was determined, and it was confirmed by electron microscopy. The evaluation of the zeta potential of the investigated complexes showed that the particles surface charge was close to the zero point. This may indicate that the positively charged PGS conjugate has completely packed the negatively charged mRNA molecules. It has been shown that the packaging of mRNA-RBD into the PGS envelope leads to increased production of specific antibodies with virus-neutralizing activity in immunized BALB/c mice. Our results showed that the proposed polycationic polyglucin:spermidine conjugate can be considered a promising and safe means to the delivery of mRNA vaccines, in particular mRNA vaccines against SARS-CoV-2.

## 1. Introduction

mRNA-based vaccines are a relatively new platform that in recent years has shown great promise for the development of potential vaccines against viral infections. The use of mRNA to create preventive vaccines has a number of attractive features: simplicity of design, low cost of production, low reactogenicity, intracellular synthesis of the target antigen, induction of both antibodies, and CD4+ and CD8+ T cell responses [1,2,3,4,5]. At the same time, the immunogenicity of mRNA vaccines in the form of a naked RNA molecule is low due to their degradation by RNases and the inefficiency of their delivery to the cells of the immune system. One of the ways to increase the immune response to an mRNA vaccine is to encapsulate it in nanoparticles. Packaging mRNA in biocompatible and biodegradable materials protects it from degradation and facilitates uptake by antigen-presenting cells [6].

A number of mRNA-based vaccines against COVID-19 are currently being developed [7,8,9]. Preclinical and clinical studies have shown that mRNA-based vaccines encoding the S protein [10] or the receptor binding domain (RBD) [11] elicit strong protective immune responses against SARS-CoV-2 with a low level of reactogenicity. Notably, at the moment, pharmaceutical companies Moderna/NIH and Pfizer/BioNTech have already proven the effectiveness of their mRNA vaccines in clinical trials and are at the stage of preparing their registration.

An important and not fully solved problem of mRNA vaccines development is their delivery. Various forms of carriers are used for mRNA delivery, including lipids, polymer and polypeptide systems, dendrimers, gold nanoparticles, and hybrid systems [12,13]. Lipid nanoparticles are currently one of the most commonly used mRNA delivery vehicles. Almost all developers of mRNA vaccines against SARS-CoV-2, including Moderna/NIH and Pfizer/BioNTech, encapsulate RNA molecules in lipid nanoparticles [14].

The main problem of mRNA delivery using liposomes is associated with the nature of lipids. In particular, positively charged liposome particles can bind to negatively charged proteins and nucleic acids and adhere to cell surfaces, which can destabilize the plasma membrane and may cause side effects in the person being vaccinated [15,16,17,18]. Different modifications of liposomes components may be a possible solution [5]; however, this increases the cost of production of lipid nanoparticles and complicates their composition, which can lead to undesirable consequences. Another problem with lipid nanoparticles is that they are sensitive to freezing and thawing during lyophilization, which makes them difficult to use for mass vaccination [19,20].

The main task of the work was to evaluate the effectiveness of one of the alternative methods of mRNA delivery based on the use of the polyglucin:spermidine conjugate. We investigated the polyglucin:spermidine conjugate as a carrier of an mRNA-RBD vaccine. Data on the ability of these complexes to induce a humoral immune response in laboratory animals are presented. 

## 2. Materials and Methods 

### 2.1. mRNA Synthesis

Two genetic constructs encoding the target genes and T7 promoter were used as a template for mRNA synthesis. The first of these was the pVAX-RBD plasmid. To construct the plasmid, a fragment of the S gene corresponding to RBD (320–542 aa) was used (GenBank MN908947). Optimization of the codon composition of the sequence was carried out using the GeneOptimizer program (https://www.thermofisher.com/ru/en/home/life-science/cloning/gene-synthesis/geneart-gene-synthesis/geneoptimizer.html). To ensure efficient transport of the synthesized protein from the cell, a fragment encoding the signal sequence MMRTLILAVLLVYFCATVHC was added to the 5’-end of the gene. It is a hybrid of two signal sequences: luciferase and fibroin proteins. The synthesis of the projected nucleotide sequence as part of the pVAX vector was carried out at OOO DNA-synthesis (Russia). The second construct was the commercial plasmid phMGFP (Thermo Fisher Scientific, Waltham, MA, USA), which encodes the green fluorescent protein (GFP) gene. This plasmid was used as a template for the synthesis of mRNA-GFP to control the correctness of the procedure for capping and polyadenylation of RNA, as well as a negative control for immunization of animals.

The synthesis of mRNA was carried out using linearized DNA templates and T7 RNA polymerase (SibEnzyme, Novosibirsk, Russia) according to the manufacturer’s protocol. The reaction mixture included 1 μg of the linearized template, T7 polymerase with buffer, a mixture of ribonucleotides in which uridine was replaced by pseudouridine, an RNase inhibitor, and nuclease-free water. The mixture was incubated for one hour at 37 °C. Polyadenylation and capping were performed using the ScriptCap™ m7G CappingSystem (CELLSCRIPT, Madison, WI, USA) and A-Plus™ Poly (A) PolymeraseTailingKit (CELLSCRIPT, Madison, WI, USA) as recommended by the manufacturer. Purification of mRNA was performed on Monarch^®^ TotalRNAMiniprepKit columns (New England Biolabs, Ipswich, MA, USA).

### 2.2. Preparation of the Polyglucin:Spermidine Conjugate

Synthesis of polysaccharide conjugate was performed as described earlier in [21,22,23]. Briefly, polyglucin (dextran 40,000) was incubated with sodium periodate (40 molecules of sodium periodate per 1 molecule of dextran) for one hour and desalted by gel filtration on Sephadex G-25 in 50 mM carbonate buffer (pH 8.6). Activated polyglucin was combined with spermidine (at the rate of 15 molecules of spermidine per 1 molecule of dextran) and the mixture was incubated for 12 h at room temperature. Sodium borohydride was added to the mixture (80 molecules of borohydride per one molecule of dextran) and stirred for 2 h at room temperature. Unreacted components were removed by gel filtration on Sephadex G-25. As a result, a polyglucin:spermidine (PGS) conjugate was obtained, in which there were 15 spermidine molecules per one dextran molecule. The conjugate structure was confirmed by ^1^H NMR spectroscopy.

To form complexes for immunizing animals, mRNA was mixed with PGS in a charge ratio of 1:5, which corresponds to a weight ratio of 1:15. It was mixed with 100 μL of a solution with a concentration of 0.06 mg/mL mRNA-RBD and 100 μL of a solution containing 0.93 mg/mL PGS, gently stirred for 2–3 s, then incubated at room temperature for one hour. mRNA-PGS complexes were analyzed by electrophoresis in 2% agarose gel and dynamic light scattering by electron microscopy.

### 2.3. Dynamic and Electrophoretic Light Scattering

The hydrodynamic size of nanoparticles formed by mRNA and PGS and their kinetic layer potential (zeta potential) were determined by dynamic and electrophoretic light scattering methods using a Zetasizer NanoZSPlus (Malvern Instruments; Malvern, UK). ZEN0040 cuvettes were used to measure the size, and DTS1070 was used to measure the possibility of the kinetic layer of nanoparticles. All measurements were made in triplicate at 25 °C. The mean particle size parameter, polydispersity index (PdI) parameter, and mean zeta ability were used.

### 2.4. Electron Microscopy

To assess the size and shape of the mRNA-RBD-PGS complexes, the suspension was applied to electron microscopy copper grids covered with a carbon stabilized formvar film. The preparations were stained with a 2% aqueous solution of uranyl acetate. The study was carried out using a JEM-1400 electron microscope (Jeol, Tokyo, Japan). Image acquisition, image analysis and processing were performed using a Veleta digital camera (EMSIS GmbH, Muenster, Germany) and iTEM software suite (EMSIS GmbH, Muenster, Germany).

### 2.5. Cells Transfection with mRNA-GFP

HEK293T cells were grown in six-well tissue culture plates (Costar) with modified DMEM medium (Sigma-Aldrich, St. Louis, MI, USA) supplemented with 10% FBS (HyClone) and 50 mg/mL gentamicin. Medium (250 µL) containing either 2 µg of mRNA-GFP in PGS envelope or 2 µg of mRNA-GFP in liposomes from Lipofectamine 3000 (ThermoFisher, Waltham, MA, USA) was added to the wells of a culture plate with a monolayer of 70%–80% confluent cells. The control well was injected with 2 μg of “naked” mRNA-GFP. The cell plate was placed in a 37 °C CO_2_ incubator and incubated for 4 h. After that, the culture medium was replaced with a fresh one and the incubation continued for 24 h. The results were visualized using an Olympus CKX53 microscope or flow cytometry. To determine GFP expression levels, 20,000 events per sample gated on single cells were acquired on a Ze5 flow cytometer (Bio-Rad Laboratories Inc., Hercules, CA, USA) and analyzed using FlowJo software.

### 2.6. Mice Immunization

Work with animals was carried out according to the “Guide for the Care and Use of Laboratory Animals”. The protocols were approved by the Institutional Animal Care and Use Committee (IACUC) affiliated with the State Research Center of Virology and Biotechnology “Vector” (Permit Number: SRC VB “Vector”/10-09.2020). To assess the immunogenicity of mRNA-RBD-PGS, female BALB/c mice weighing 16–18 g were used. Mice were divided into the following four groups with each group consisting of six animals: Group 1 (mRNA-RBD-PGS)—mice immunized with mRNA-RBD-PGS (10 µg RNA/100 µL normal saline); Group 2 (mRNA-RBD)—mice immunized with naked mRNA-RBD (10 µg RNA/100 µL normal saline); Group 3 (mRNA-GFP-PGS)—mice immunized with mRNA-GFP-PGS (10 µg RNA/100 µL normal saline); Group 4 (control)—mice immunized with normal saline (100 µL). Mice were immunized three times, intramuscularly (in the upper thigh of the hind limb) on days 0, 21, and 35. On days 34 and 48, mice were bled for serum analysis. The sera were separated from the cellular elements by centrifugation (10,000 rpm, 15 min), warmed to inactivate the complement system (30 min, 56 °C).

### 2.7. Serum ELISA

The eukaryotic proteins RBD and S-trimer were used as antigens for ELISA, which were kindly provided by the laboratory of immunochemistry of the FBSI SSC VB Vector of Rospotrebnadzor (Russia). The RBD protein (1 μg/mL) and S-trimer (1 μg/mL) were adsorbed in the wells of a 96-well plate in PBS (Greiner Bio One GmbH, Frickenhausen, Germany) at 4 °C for 12 h, then washed with PBST and blocked with 1% casein solution in wash buffer for 60 min at room temperature. After that, serum samples were added in a threefold serial dilution, starting at 1:50, and incubated for 60 min at 37 °C. After washing, rabbit anti-mouse IgG antibodies conjugated with horseradish peroxidase (Sigma-Aldrich, St. Louis, MO, USA) were added and incubated for 60 min at 37 °C. The plate was washed and a solution of TMB substrate (Amresco LLC, Solon, OH, USA) was added to it. After stopping the reaction with the stop solution, the optical density was measured at a wavelength of 450 nm using an ELISA reader (ChroMate Awareness technology Inc., Palm City, FL, USA). The graphs were built using GraphPad Prism 6.0 and Excel 2016.

### 2.8. Neutralizing Activity of Immune Sera

The work used the coronavirus 2019-nCoV strain nCoV/Victoria/1/2020 (State collection of pathogens of viral infections and rickettsioses FBSI GNTS BV “Vector” Rospotrebnadzor, RF). The working dose of the virus was 100 CPD50. To assess the effectiveness of the neutralizing activity of the sera, their serial two-fold dilutions were prepared starting from a dilution of 1:10 to 1:1000. For dilution of sera, we used MEM medium with glutamine and the addition of antibiotics (penicillin 100 U/mL, streptomycin-100 μg/mL). A mixture of serum dilutions and a working virus dilution was prepared in equal volumes. The mixture was incubated for 1 h at room temperature, then added to the wells of a 96-well plate with a monolayer of Vero E6 cell culture. The plates were incubated for four days at 37 °C, 5% CO_2_. To stain the cells, 150 μL of 0.2% gentian violet solution was added to each well of the plate (1 g of gentian violet was dissolved in 20 mL 96% ethanol, 120 mL 40% formalin, and 350 mL Hanks solution were added). After 30 min, the liquid from the wells was removed and washed with water. The results were recorded visually. Any specific damage to the cell culture in the well was counted as the cytopathic effect of the virus (CPV). The neutralizing activity of the serum of the immunized animals was assessed by the titer (dilution) of the serum, at which point the protection in 50% of the wells with the cell culture against the cytopathic action of the virus was recorded. The titer of neutralizing antibodies was calculated using the Reed–Muench formula [24].

### 2.9. Cell Viability Assay (MTT)

Cytotoxicity of mRNA-RBD-PGS and mRNA-RBD-Lipofectamine 3000 complexes were evaluated by MTT assay [25]. HEK293 cells (1 × 10^5^ cells/mL) in 100 μL of culture media were seeded in 96-well plates and incubated overnight. The next day, mRNA-RBD-PGS and mRNA-RBD-Lipofectamine 3000 complexes were added to the cells and incubated for 72 h. After 72 h of incubation of cells with the tested complexes, 20 μL of MTT solution (5 mg/mL) was added to each well and incubated for 2 h in a CO_2_ incubator. After incubation, the medium was aspirated, and dimethyl sulfoxide (50 μL/well) was added to stop the reaction. The optical density was quantified in Varioskan LUX multimode microplate reader (Thermo Fisher Scientific, Waltham, MA, USA) at 570 nm wavelength. The cell viability in the presence of complexes was calculated using the formula: (optical density of the test wells/optical density of the control wells) × 100%.

## 3. Results 

### 3.1. Characterization of the Synthesized mRNA

**Figure 1:**
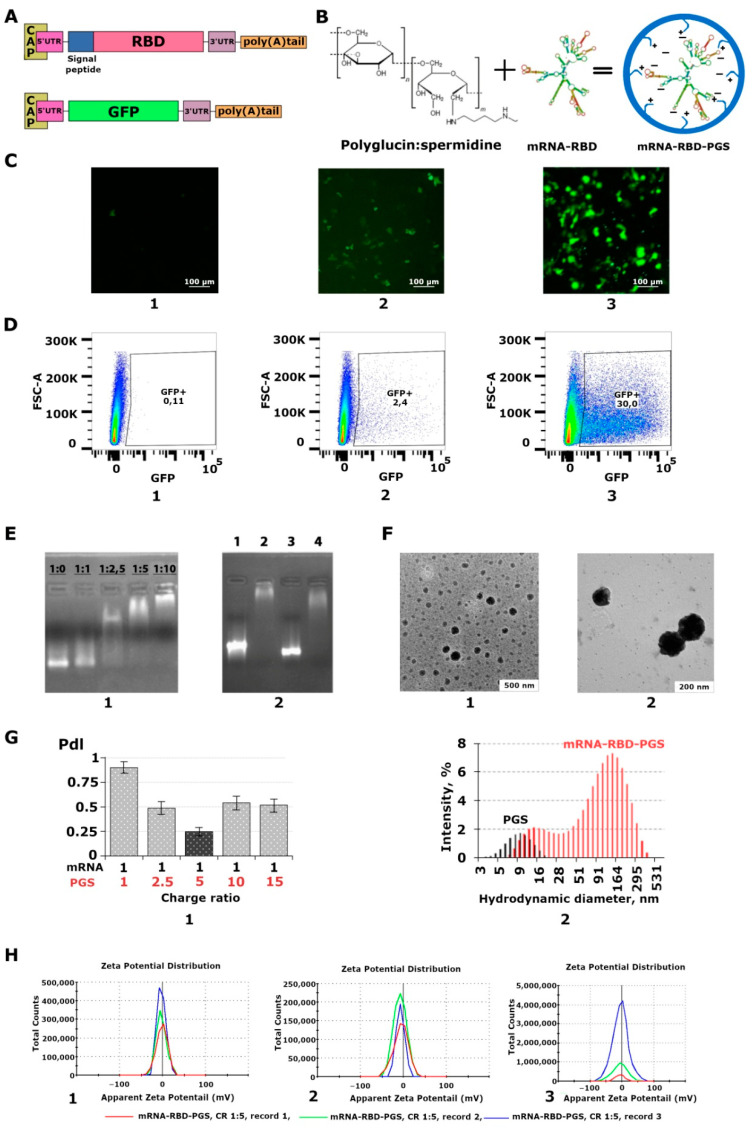
Preparation and characterization of mRNA and polyglucin:spermidine conjugate (PGS) complexes. (**A**) Schematic representation of mRNA-RBD, and mRNA-GFP. mRNA has a cap, poly (**A**) tail, 3’ and 5’ UTRs, and ORF encoding receptor binding domain (RBD) or green fluorescent protein (GFP). mRNA-RBD additionally contains a signal peptide to increase the secretion of the synthesized protein. (**B**) Self-assembly of mRNA-RBD-PGS or mRNA-GFP-PGS complexes. The chemical formula of the polyglucin:spermidine conjugate and the hypothetical structure of mRNA and mRNA-PGS complexes is presented. (**C**) Transfection of HEK293T cell culture with: (1) mRNA-GFP, (2) mRNA-GFP-PGS, and (3) mRNA-GFP-Lipofectamine 3000. Results were visualized with an Olympus CKX53 microscope 24 h after transfection. (**D**) Flow cytometry analysis of GFP expression in transfected cells: (1) mRNA-GFP, (2) mRNA-GFP-PGS, and (3) mRNA-GFP-Lipofectamine 3000. Results were visualized 24 h after transfection. (**E**) Characterization of mRNA-RBD-PGS complexes: (1) Electrophoresis in 2% agarose gel. Selection of the mRNA:PGS ratio. The charge ratios are indicated above the track, (2) Electrophoresis in 2% agarose gel. Control of the degree of coverage of mRNA with PGS conjugate at N:P equal to 1:5 before immunization. mRNA encapsulated with PGS loses its mobility in an electric field. 1—mRNA-RBD, 2—mRNA-RBD-PGS, 3—mRNA-GFP, 4—mRNA-GFP-PGS. (**F**) Electron micrograph of mRNA-RBD-PGS complexes at N:P equal to 1:5, (1, 2). (**G**) Dynamic light scattering: (1) polydispersity index (PdI), (2) size distribution profiles of nanoconstructions formed by mRNA-RBD upon complexation with PGS. (**H**) Measurement of the zeta potential of the resulting complexes at N:P equal to 1:5 in three different series (1,2,3).

The structures of mRNA-RBD and mRNA-GFP are shown schematically in Figure 1A. mRNAs were obtained using linearised plasmids pVAX-RBD and phMGFP as templates and T7 polymerase. RNA capping and polyadenylation were performed using appropriate enzymes (see the Materials and Methods section). The level of polyadenylation was judged by the change in RNA mobility using electrophoresis in 2% agarose gel. The resulting RNA was compared with a negative control without polyadenylation; a difference was observed in the bands from 100 to 200 nucleotides. Control of mRNA-RBD capping was carried out by parallel preparation of mRNA-GFP under the same conditions. To confirm the expression of mRNA encoding GFP, HEK293T cells were transfected using Lipofectamine 3000 mRNA (ThermoFisher) as a carrier. The data presented in Figure 1C,D confirmed that mRNA-GFP is functional and mediates the synthesis of the GFP protein in HEK293T cells. Transfection efficiency with Lipofectamine 3000 was 30% and with PGS, 2.4%.

### 3.2. Characterization of the mRNA-PGS Complex

A schematic representation of the preparation of RNA and PGS complexes is shown in Figure 1B. To form the complexes, RNA was mixed with PGS in certain charge ratios. The formation of mRNA:PGS complexes was investigated to determine the relationship of stable association between polymer and mRNA, as well as the biophysical characteristics of the complexes. Complexation was assessed using the PGS/mRNA charge ratio (CR), which is related to the PGS/mRNA molar ratio (MR). CR is the ratio of the total positive charge of the polymer (positively charged peripheral amino groups in the cationic polymer) to the total negative charge of mRNA (phosphate groups) in the sample, which was calculated as follows:

(1) $$CR=\frac{C_{+}\times Z_{+}}{C_{-}\times Z_{-}}=MR\times \frac{Z_{+}}{Z_{-}},$$

where $C_{+}$ is the final concentration of the cationic polymer in the sample, $Z_{+}$ is the charge of a single cationic polymer molecule, $C_{-}$ is the final concentration of nucleic acid in the sample, and $Z_{-}$ is the charge of a single nucleic acid molecule [26].

Based on the charge of RNA, which corresponds to its length and nucleotide composition, the molar mass of the polymer required to neutralize the negative charge of the nucleic acid was calculated, considering that there are 45 cations per PGS molecule. To select the optimal ratio of mRNA: PGS for the formation of complexes, a series of samples with different charge ratios (N:P) of RNA to polymer were obtained, where (N) is the negative charge of RNA, and (P) is the positive charge of the cationic polymer. The following N:P for RNA:PGS were taken: 1:1, 1:2.5, 1:5, and 1:10, which corresponds to the ratio by weight of 1:3, 1:8, 1:15, and 1:30. So, for example, in a 1: 1 charge ratio, 1 μg of RNA was taken and mixed with 3 μg of cationic polymer in equal volumes.

The initial assessment of mRNA coverage with PGS was the mobility of mRNA-PGS in an electric field during electrophoresis in 2% agarose gel, the degree of which depended on the degree of coverage. It can be seen in Figure 1E1 that with an increase in N:P there was a decrease in the electrophoretic mobility of mRNA-PGS complexes in the gel, which indirectly indicates the formation of complexes.

Furthermore, the obtained complexes were characterized by dynamic light scattering and zeta potential measurements. Analysis using the dynamic light scattering method (Figure 1G1) showed that for all N:P (except 1:5), the polydispersity index (PdI) of the corresponding complexes was high and varied from 0.48 ± 0.07 to 0.90 ± 0.05, which indicates the formation of nanoparticles of different sizes from 20 to 800 nm. When the mRNA:PGS charge ratio was 1:5, the most monodispersed suspension of nanoparticles was formed (PdI = 0.25 ± 0.04). The average hydrodynamic diameter of mRNA:PGS at N:P equal to 1:5 was 164 ± 20 nm. The particle distribution profile (Figure 1G2) shows two peaks with maxima at 13 and 164 nm. The first probably corresponds to the residual amount of PGS, the second to the resulting mRNA:PGS complex.

Evaluation of the zeta potential of the complexes in three different series showed that the value of the zeta potential of the particles at mRNA:PGS charge ratio equal to 1:5 was −4.34 ± 2.42 mV, which is in agreement with our calculations (Figure 1H). The presence of a weak charge may indicate that the positively charged cationic polymer almost completely packed the negatively charged mRNA molecules. To confirm the formation of nanoparticles, electron microscopy was also carried out (Figure 1F1,2). The particle size at N:P equal to 1:5 was 100–200 nm, which corresponds to the average size of the virus.

Thus, for the coverage of the vaccine, we chose an RNA:PGS charge ratio of 1:5. A comprehensive study showed that this ratio provides coverage of mRNA with a cationic polymer, as evidenced by the absence of charge on the particles when measuring the zeta potential and a decrease in electrophoretic mobility in an agarose gel. This polymer ratio provides the formation of nanoparticles which was confirmed by dynamic light scattering and electron microscopy.

Before immunization of animals, preparative synthesis of mRNA-RBD-PGS and mRNA-GFP-PGS was carried out. Electrophoretic analysis of the resulting complexes (Figure 1E2) indirectly confirmed their assembly, and the mobility in the gel corresponded to the above calculations of the RNA:PGS charge ratio of 1:5.

### 3.3. Cytotoxicity of the mRNA-RBD-PGS Complexes 

**Figure 2:**
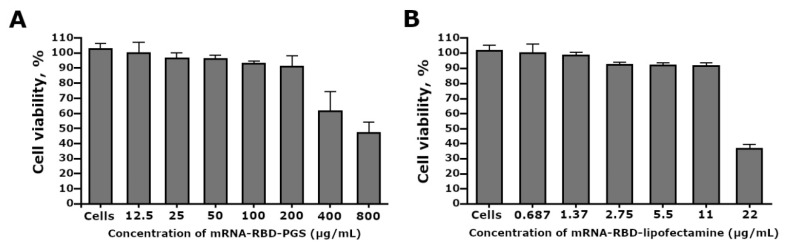
Estimation of HEK293 cell viability treated with mRNA-RBD-PGS (**A**) and mRNA-RBD-Lipofectamine 3000 (**B**) by MTT assay. The results are expressed as means ± SD of triplicate experiments.

We investigated the effects of mRNA-RBD-PGS complexes and mRNA-RBD-Lipofectamine 3000 liposomes on cell viability of HEK293T cells by using the MTT assay. The MTT test showed that incubation of HEK293 cells with the mRNA-RBD-PGS complexes in the concentration range from 800 to 12.5 μg/mL, the proportion of viable cells relative to the control ranged from 51.3 ± 12.8% to 98.5 ± 3.3% respectively (Figure 2A). Untreated HEK293cells control was 99.1 ± 2.4%. When mRNA-RBD-Lipofectamine 3000 was added to the cell culture in the concentration range from 22 to 0.687 μg/mL, the percentage of cell viability ranged from 36.8 ± 2.6% to 99.9 ± 4.8% respectively (Figure 2B). The CC_50_ values of mRNA-RBD-PGS and mRNA-RBD-Lipofectamine 3000 against HEK293 cells were 625 µg/mL and 20.4 µg/mL, respectively.

### 3.4. Mice Immune Response to Vaccine

To assess the immunogenicity of the mRNA-RBD-PGS and mRNA-RBD vaccine constructs, mice were immunized three times on days 0, 21, and 35. Five and seven weeks after the start of immunization, sera were taken from the mice, which were tested in ELISA for the presence of antibodies specifically recognizing RBD and S-trimer, and the ability of the sera to neutralize the virus was also examined. Sera from mRNA-GFP-PGS immunized mice and saline were used as controls.

**Figure 3:**
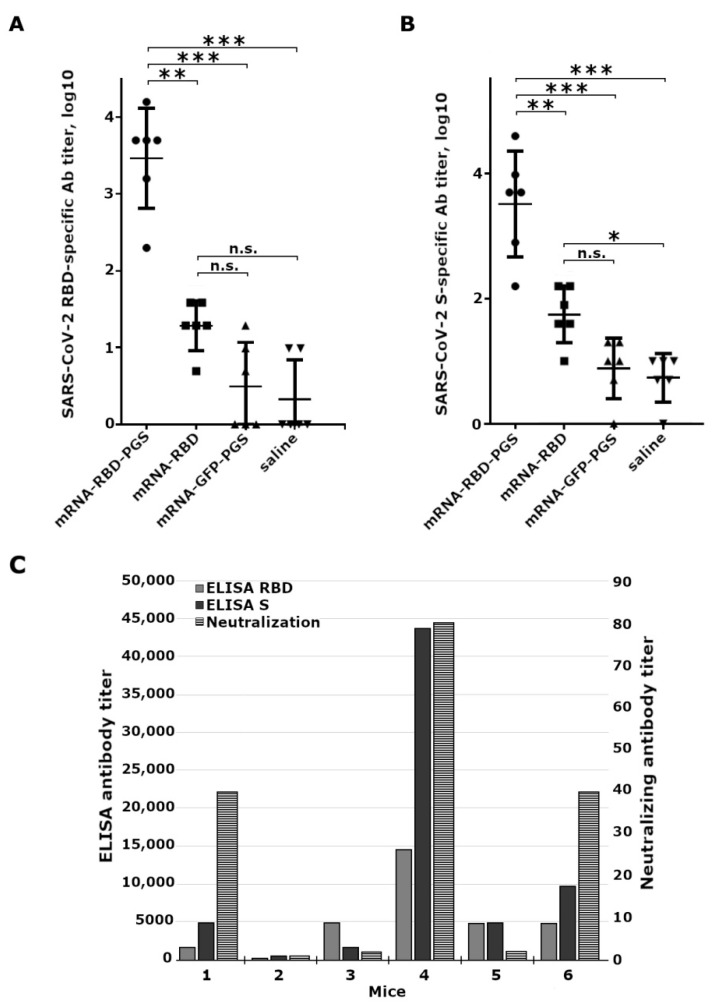
Humoral responses to SARS-CoV-2 antigens in immunized BALB/C mice. Mice were immunized intramuscularly with naked mRNA-RBD, complexes of mRNA with conjugate polyglucin:spermidine (mRNA-RBD-PGS or mRNA-GFP-PGS), or saline, three times at 0, 21, and 35 days (10 µg mRNA per mouse). Sera samples were collected on mice 48 days after the first immunization. (**A**) The SARS-CoV-2 RBD specific IgG antibody titers and (**B**) the SARS-CoV-2 S specific IgG antibody titers were determined by ELISA. Statistical analysis was performed using GraphPad Prism 6.0 software. Data are presented as mean ± standard error of the mean. Significance was calculated using two-way analysis of variance with multiple comparison tests (n.s., not statistically significant; * *p* < 0.05, ** *p* < 0.01, *** *p* < 0.001). (**C**) Data of ELISA and virus neutralizing activity of sera from mRNA-RBD-PGS immunized mice. Abscissa axis: 1–6—mouse number. The left ordinate axis is the reciprocal antibody titer obtained by ELISA, the right ordinate axis is the reciprocal value of the neutralizing serum titer: grey bar—anti-RBD specific antibodies, white bar—anti-RBD specific antibodies, black bar—virus-neutralizing antibodies.

The ELISA data showed that two weeks after the second immunization there was a slight increase in the titer of specific antibodies, but no significant differences were found with negative control groups. Two weeks after the third immunization in the mRNA-RBD-PGS group, a significant increase (100–1000 times) in the titer of specific antibodies to both the RBD and S-trimer were observed in comparison with the sera of mice immunized with mRNA-RBD, mRNA-GFP-PGS, and saline (Figure 3A,B).

Analysis of the neutralizing activity of the sera was carried out using the live SARS-CoV-2 virus of the nCoV/Victoria/1/2020 strain and the Vero E6 cell culture. Virus neutralizing antibodies were detected only in the group of animals immunized with mRNA-RBD-PGS, and serum of animals with high-titers of anti-RBD antibodies possessed virus-neutralizing activity (Figure 3C). 

## 4. Discussion

The use of mRNA to create preventive vaccines has a number of attractive features: simplicity of design, low cost of production, low reactogenicity, intracellular synthesis of the target antigen, induction of both antibodies, and CD4+ and CD8+ T-cell responses [3,5]. At the same time, the immunogenicity of mRNA vaccines in the form of a naked RNA molecule is low due to their degradation by RNases and the inefficiency of their delivery to cells of the immune system. One of the ways to increase the immune response to an mRNA vaccine is to encapsulate it in nanoparticles consisting of biodegradable cationic polymers and/or liposomes or lipid nanoparticles (LNP) which protect mRNA from degradation and ensure its delivery to antigen-presenting cells.

To deliver mRNA by liposomes, LNPs obtained using cationic lipids containing tertiary or quaternary amines are usually used [13]. There are many liposomal formulations, but they all have a number of disadvantages associated, primarily with the lipids nature. Thus, positively charged liposome particles can bind to negatively charged proteins and nucleic acids and adhere to cell surfaces, which can destabilize the plasma membrane and cause toxic reactions in vaccinated individuals [27,28,29]. To solve this problem, ionizable lipids can be used [5]. However, this complicates the composition of lipid nanoparticles and increases the cost of their production. Another problem with lipid nanoparticles is that they need to be transported and stored in a continuous cold chain process, which is a difficult logistical challenge [19]. Therefore, the development of new delivery systems providing thermal stability of mRNA vaccines is gaining more and more interest [30]. Work is underway to obtain thermostable LNPs. For example, Dolgin reported that the Chinese company whose COVID-19 RNA vaccine is currently in human trials has focused on the quality and purity of LNP. Their vaccine remains effective for up to one week at room temperature [31]. However, it should be noted that LNP is a multicomponent structure, its stabilization requires the use of additional components, and such liposomes can cause additional undesirable reactions to the administration of the developed vaccine.

In our work, we obtained an experimental mRNA vaccine encoding the receptor binding domain of the SARS-CoV-2 spike protein. RBD is considered one of the main immunogens in the development of a vaccine against SARS-CoV-2, since in that domain the main virus-neutralizing epitopes are concentrated [32,33,34,35,36,37,38].

To package mRNA, a polycationic polymer polyglucin:spermidine conjugate (PGS) was used. The choice of PGS was due to the fact that we had previously successfully tested it for the delivery of DNA vaccines [21,22,23,39]. PGS was included in the candidate HIV-1 vaccine CombiHIVvac, which passed the first phase of clinical trials [40,41] which demonstrated the safety of the vaccine. The polyglucin envelop has also been shown to protect yeast dsRNA from degradation by serum nucleases [21].

Unlike liposomes, PGS contains only two components, polyglucin and spermidine, which distinguishes it from liposomal nanoparticles. PGS allows nucleic acid to be lyophilized and stored for extended periods at positive temperatures. The PGS conjugate is a component of the HIV-1 DNA vaccine and its safety has been confirmed in the preclinical studies and Phase I clinical trials [40]. It was demonstrated that DNA vaccine encapsulated with PGS as part of the preparation is retained without loss of specific activity for at least two years at 4 °C.

It is attractive that the components of PGS are biodegradable and safe for humans. Polyglucin, a glucose polymer with a molecular weight of 40,000, is non-toxic to humans and is a licensed plasma-substituting drug of hemodynamic action that restores the volume of circulating blood [42]. Spermidine is a naturally occurring polyamine found in all living organisms; it is critical for maintaining cellular homeostasis and is involved in numerous biological processes, including cell growth and proliferation, DNA and RNA stabilization, enzymatic modulation, and translation regulation [43,44,45,46]. In addition, the low cost of vaccine components and the possibility of lyophilization and storage of the PGS conjugate at +4 °C provide additional technological advantages in the production and transportation of the vaccine.

During the work, the conditions for the self-assembling of mRNA-PGS complexes were optimized, including the selection of the mRNA:PGS charge ratios for the formation of complexes. Their mobility in agarose gel, sizes, and surface charge were determined (Figure 1E). It was shown that the most monodisperse suspension of nanoparticles was formed at the mRNA:PGS charge ratio equal to 1:5. The average hydrodynamic particle diameter was 164 ± 20 nm (Figure 1G2), which was confirmed by electron microscopy. The evaluation of the zeta potential of the investigated complexes showed that its value was close to the zero point. The weak charge of the complexes may indicate that the positively charged PGS conjugate has completely packed the negatively charged mRNA molecules. In addition, a weak charge may further indicate the complexes safety, since there is evidence that cationic and anionic nanoparticles are generally more cytotoxic than neutral ones [47]. Cytotoxicity analysis of our preparation on HEK293 cell culture showed that mRNA-RBD-PGS is 30 times less toxic than mRNA-RBD coated with Lipofectamine 3000 (Figure 2A,B).

The mRNA encoding GFP in the PGS envelope had weak transfection potential compared to lipofectamine during in vitro transfection of HEK 293T cells (Figure 1F), indicating the low affinity of the mRNA-GFP-PGS complexes for the cell membrane. This may be due to the physicochemical properties of the complexes, and in particular, due to the low surface charge of the mRNA-GFP-PGS particles as demonstrated by experiments on evaluating the zeta potential. At the same time, liposomes from phospholipids more efficiently penetrate cells, in particular, due to the fact that they have a high surface charge, which, however, is one of the reasons for the toxicity of liposomes (Figure 2) [27]. By adhering to cell surfaces, liposomes can destabilize the cell membrane and cause toxic reactions in vaccinated people [18]. Despite the weak transfecting activity, mRNA-RBD-PGS induces strong humoral immune response in laboratory animals. Apparently, in the case of in vivo mice immunization, the mRNA-RBD-PGS complex penetrates into antigen-presenting cells by endocytosis due to its size, which is comparable to the average size of viral particles (100–200 nm). Most likely this was the main reason for the increase in the immunogenicity of mRNA-RBD-PGS versus naked mRNA (mRNA-RBD) in our experiments. Indeed, particle size was known to be an important parameter influencing the recognition and endocytosis of vaccine constructions by APCs [48].

When assessing the immunogenicity of mRNA-RBD, we were interested in its ability to induce virus-specific antibodies, which are one of the main indicators of vaccine protection. Three-fold intramuscular immunization of mice with naked mRNA-RBD (10 μg per mouse), obtained using pseudo-uridine, did not lead to a significant increase in the titer of specific antibodies compared to the negative control (mRNA-GFP-PGS) (Figure 1A,B). Three-fold immunization of mice with mRNA-RBD in the PGS envelope led to the induction of specific antibodies, the titers of which in ELISA were significantly higher compared to mice immunized with both mRNA-GFP-PGS (negative control, *p* ≤ 0.01) and naked mRNA-RBD (*p* ≤ 0.05) (Figure 3A,B), and serum of animals with high-titers of anti-RBD antibodies possessed virus-neutralizing activity (Figure 3C).

Apparently, the complexation of mRNA with PGS promotes its uptake by antigen-presenting cells and increases the immune response to the target antigen, in comparison with naked mRNA. In addition, the PGS envelope can protect mRNA from degradation by nucleases, as a result of which the mRNA lifetime is increased, which eventually leads to a prolongation of the immune response.

In this article, we did not optimize the structure of mRNA and its regulatory regions, which can increase the expression of target genes. The main task of the work was to evaluate the effectiveness of one of the alternative methods of mRNA delivery based on the use of the polyglucin:spermidine conjugate.

## 5. Conclusions

In this study, we investigated the polyglucin:spermidine conjugate as a carrier of an mRNA-RBD vaccine. The optimal conditions for the self-assembly of mRNA-PGS complexes were selected, and the sizes and surface charge of the complexes were determined. It was shown that the packaging of mRNA in the PGS envelope leads to a significant increase in their immunogenic properties and to the induction of virus-specific antibodies in BALB/c mice. Our results show that the proposed polycationic polymer can be considered a promising and safe means to deliver mRNA vaccines, in particular mRNA vaccines against SARS-CoV-2.

## Data Availability

No new data were created or analyzed in this study. Data sharing is not applicable to this article.
